# The dispersal between Amazonia and Atlantic Forest during the Early Neogene revealed by the biogeography of the treefrog tribe Sphaenorhynchini (Anura, Hylidae)

**DOI:** 10.1002/ece3.8754

**Published:** 2022-04-01

**Authors:** Elvis Almeida Pereira, Karoline Ceron, Hélio Ricardo da Silva, Diego José Santana

**Affiliations:** ^1^ Laboratório de Herpetologia Departamento de Biologia Animal Universidade Federal Rural do Rio de Janeiro Rio de Janeiro Brazil; ^2^ 54534 Mapinguari ‐ Laboratório de Biogeografia e Sistemática de Anfíbios e Répteis Universidade Federal de Mato Grosso do Sul Campo Grande Brazil; ^3^ Laboratório de Genética e Biodiversidade Universidade Federal do Oeste do Pará Santarém Brazil; ^4^ Departamento de Biologia Animal Universidade Estadual de Campinas (UNICAMP) São Paulo Brazil

**Keywords:** dispersal, *Gabohyla*, hatchet‐faced tree frog, lime Tree Frogs, phylogeny, short‐snouted green tree frogs, *Sphaenorhynchus*, zoogeography

## Abstract

The Amazonia and the Atlantic Forest, separated by the diagonal of open formations, are two ecoregions that comprise the most diverse tropical forests in the world. The Sphaenorhynchini tribe is among the few tribes of anurans that occur in both rainforests, and their historical biogeographic have never been proposed. In this study, we infer a dated phylogeny for the species of the Sphaenorhynchini and we reconstructed the biogeographic history describing the diversification chronology, and possible patterns of dispersion and vicariance, providing information about how orogeny, forest dynamics and allopatric speciation affected their evolution in South America. We provided a dated phylogeny and biogeography study for the Sphaenorhynchini tribe using mitochondrial and nuclear genes. We analyzed 41 samples to estimate the ancestral areas using biogeographical analysis based on the estimated divergence times and the current geographical ranges of the species of Sphaenorhynchini. We recovered three characteristic clades that we recognize as groups of species (*S*. *lacteus*, *S*. *planicola*, and *S*. *platycephalus* groups), with *S*. *carneus* and *G*. *pauloalvini* being the sister taxa of all other species from the tribe. We found that the diversification of the tribe lineages coincided with the main climatic and geological factors that shaped the Neotropical landscape during the Cenozoic. The most recent common ancestor of the Sphaenorhynchini species emerged in the North of the Atlantic Forest and migrated to the Amazonia in different dispersion events that occurred during the connections between these ecoregions. This is the first large‐scale study to include an almost complete calibrated phylogeny of Sphaenorhynchini, presenting important information about the evolution and diversification of the tribe. Overall, we suggest that biogeographic historical of Sphaenorhynchini have resulted from a combination of repeated range expansion and contraction cycles concurrent with climate fluctuations and dispersal events between the Atlantic Forest and Amazonia.

## Graphical abstract

We suggest that phylogeographic patterns of Sphaenorhynchini have resulted from a combination of repeated range expansion and contraction cycles concurrent with Quaternary climate fluctuations and stochastic dispersal events between the Atlantic Forest and Amazonian

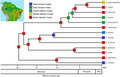

## 1 INTRODUCTION

The Neotropical region is the most diverse region on Earth, having from recent to old geological features, covering a wide range of geological and geomorphological formations (Hoorn et al., 2010; Saadi, 1995) and the speciation time among organisms in this region has been widely debated (e.g., Batalha‐Filho et al., 2012; de Sá et al., 2019; Fouquet, Loebmann, et al., 2012; Fouquet, Recoder, et al., 2012; Hoorn, Wesselingh, Hovikoski, et al., 2010; Rull, 2008, 2011a, 2011b). Different hypotheses were suggested for the origin and maintenance of biodiversity in this region, such as the isolation of South America, the Andean uplift, the formation of the Isthmus of Panama land bridge, and the Quaternary climatic fluctuations (Batalha‐Filho et al., 2012; Fouquet, Loebmann, et al., 2012; Fouquet, Recoder, et al., 2012; Fouquet, Recoder, et al., 2012; Hoorn & Wesselingh, 2010; Paz et al., 2019). The original idea, considering the richness of tropical species as a result of a long‐term process in stable environments, was abandoned with the refugia hypothesis, and most of the speciation was attributed to Quaternary events (Haffer, 1969, 2001). More recently, growing evidence of pre‐Quaternary differentiation has accumulated, attributing tectonic, eustatic, and orogenic events (Geurgas et al., 2008; Ribas et al., 2009; Rull, 2008), or rivers as barriers to gene flow (Gascon et al., 2000; Passoni et al., 2008; Pellegrino et al., 2005).

The Amazonia and Atlantic Forest ecoregions in South America (Ab’Saber, 1977; Dinerstein et al., 2017) comprise the most diverse tropical forests in the world and are separated by the diagonal of open formations (Prado & Gibbs, 1993; Silva et al., 2004), which acts as a climatic barrier to species migration between these forested regions (Costa, 2003; Mori et al., 1981; Por, 1992). The “diagonal of open formations,” also known as “the main South American disjunction” (Brieger, 1969), began to emerge during the Oligocene (Hoorn, Wesselingh, ter Steege, et al., 2010; Perret et al., 2013; Sobral‐Souza et al., 2015). At the end of the Miocene (11–5 Mya), the increase in aridity was responsible for the rapid expansion of savanna vegetation and the separation of forests, remotely continuous, in two separate regions, the Amazonia to the West and the Atlantic Forest to the East, fully formed in the Pleistocene (2 Mya) (Arruda et al., 2018; Costa et al., 2018; Roig‐Juñent et al., 2006; Sobral‐Souza et al., 2015; Wesselingh & Salo, 2006). Currently, this dry corridor comprises the Chaco, the Pantanal, the Cerrado, and the Caatinga, Neotropical savannas, and seasonally dry forests (Ab’Saber, 1977; Sobral‐Souza et al., 2015). Such separation means that both forests have few species or species groups in common (e.g., *Lithobates palmipes*, *Rhinella margaritifera* group; *Pristimantis conspicillatus* group), and several clades endemic to each region (Fouquet, Recoder, et al., 2012). However, the two forested ecoregions have already been connected during the climatic fluctuations of the Neogene and the Quaternary period, which results in conflicting biogeographic relationships between the Eastern/Western Amazonia and the North and South of the Atlantic Forest. In addition, in relation to the animal composition, the Eastern Amazonia is more similar to the Northern Atlantic Forest and the Western Amazonia is more similar to the Southern Atlantic Forest (Cheng et al., 2013; Costa et al., 2018; Fiaschi & Pirani, 2009; Perret et al., 2006; Santos et al., 2009).

The connections between these ecoregions enabled the dispersion of several animal lineages towards the Atlantic Forest‐Amazonia during the Cenozoic (see Ledo & Colli, 2017). The patterns provided by new ideas in understanding the evolutionary relationships between the two forest ecoregions have been widely addressed in the literature, and some recent examples with different taxa indicate that these exchanges occurred at different times and in both directions, for example, for birds (Batalha‐Filho et al., 2013; Patel et al., 2011), snakes (Dal Vechio et al., 2018; Grazziotin et al., 2006; Zamudio & Greene, 1997), lizards (Prates et al., 2016; Rodrigues et al., 2014), and mammals (Costa, 2003; Pavan & Leite, 2011) in recent connections (during Pliocene and Pleistocene). It also occurred in old connections (Oligocene, middle and late Miocene) for anurans (Castroviejo‐Fisher et al., 2014; Fouquet, Loebmann, et al., 2012; Fouquet, Noonan, et al., 2012; Fouquet, Recoder, et al., 2012; Pirani et al., 2020), lizards (Prates et al., 2015, 2017), and birds (Batalha‐Filho et al., 2013).

With the Andean uplift, the neotropical landscape had multiple changes, as in this period (Paleogene–Neogene) there was a drastic change in the climate (Insel et al., 2010) and the Amazon basin and the Pebas system were formed (a large wetland of shallow lakes and swamps developed in the Western Amazonia), creating new habitats that influenced the diversification of different groups, mainly in the Amazonia ecoregion (Antonelli et al., 2009; Hoorn, 1993; Hoorn, Wesselingh, Hovikoski, et al., 2010; Hoorn, Wesselingh, ter Steege, et al., 2010). The Atlantic Forest ecoregion, located in the eastern of South America, had significant changes with the global climate transition during the Cenozoic (Carnaval & Moritz, 2008). These climatic changes in the Atlantic Forest influenced the diversification of groups in different ecoregions (Antonelli et al., 2010; Graham et al., 2010; Hughes et al., 2013) and promoted the evolution of recent lineages of diverse groups of animals and plants within the forest ecoregion (Carnaval et al., 2009; Fitzpatrick et al., 2009; Mata et al., 2009; Porto et al., 2013; Thomé et al., 2014).

Despite the limited dispersion of amphibians, some lineages are distributed in both Amazonia and Atlantic Forest regions, as the treefrog tribe Sphaenorhynchini. Within the tribe, the single species of the genus *Gabohyla* (*G*. *pauloalvini* (Bokermann, 1973)) together with 11 other species of the genus *Sphaenorhynchus* (*S*. *botocudo* Caramaschi et al., 2009, *S*. *bromelicola* Bokermann, 1966, *S*. *cammaeus* Roberto et al., 2017, *S*. *canga* Araujo‐Vieira et al., 2015, *S*. *caramaschii* Toledo et al., 2007, *S*. *mirim* Caramaschi et al., 2009, *S*. *palustris* Bokermann, 1966, *S*. *planicola* (Lutz & Lutz, 1938), *S*. *platycephalus* (Werner, 1894), *S*. *prasinus* Bokermann, 1973, and *S*. *surdus* (Cochran, 1953)), occur in the Atlantic Forest, from the State of Pernambuco to the State of Rio Grande do Sul (Araújo‐Vieira et al., 2015, 2018; Bokermann, 1966, 1973; Caramaschi et al., 2009; Cochran, 1953; da Silva et al., 2013; de Freitas et al., 2009; Lacerda & Moura, 2013; Lutz & Lutz, 1938; Melo et al., 2018; Roberto et al., 2017; Toledo et al., 2007; Werner, 1894), while the other three species have distribution associated with the Amazonia and the Orinoco basin (*S*. *carneus* (Cope, 1868), *S*. *dorisae* (Goin, 1957), and *S*. *lacteus* (Daudin, 1800)) (Benício et al., 2011; La Marca et al., 2008).

Sphaenorhynchini monophyly has always been supported (Araujo‐Vieira et al., 2020; Duellman et al., 2016; Pyron, 2014) and several morphological synapomorphies have been suggested for the tribe (Araujo‐Vieira et al., 2015; Duellman & Wiens, 1992; Faivovich et al., 2005). In addition, through recent analyzes, including morphological and molecular data (mitochondrial and nuclear genes), Araujo‐Vieira et al. (2019) conducted a phylogenetic test of Parcimony seeking to understand the internal relationships of the tribe, however, the interspecific relationships remain uncertain and the biogeographic patterns observed in this tribe have never been addressed.

Given the distribution of this clade restricted to the two largest forests ecoregions in South America, combined with the known evolutionary history between these two domains, here we report the results of a study that inferred a dated phylogenetic relationship through a Bayesian analysis among species of the Sphaenorhynchini tribe using mitochondrial and nuclear markers throughout Atlantic Forest and the Amazonia in South America. We aimed to evaluate historical biogeographical scenarios that can explain the current Sphaenorhynchini distribution. Once the Atlantic Forest harbors more species within this tribe, we hypothesized that Sphaenorhynchini originated in this ecoregion and that during the Miocene, when there was a connection between both domains, some species have dispersed to Amazonia, and subsequently vicariant into numerous widely disparate populations.

## 2 MATERIALS AND METHODS

### 2.1 Sample collection, alignment editing, and generation

We used 12 species of the genus *Sphaenorhynchus* and one of *Gabohyla* derived from previous phylogenetic studies (Araujo‐Vieira et al., 2019, 2020; Faivovich et al., 2005; Wiens et al., 2006), including the species *G*. *pauloalvini*, *S*. *botocudo*, *S cammaeus*, *S*. *canga*, *S*. *caramaschi*, *S*. *carnaeus*, *S*. *dorisae*, *S*. *lacteus*, *S*. *mirim*, *S*. *planicola*, *S*. *platycephalus*, *S*. *prasinus*, and *S*. *surdus*. All sequences are available at Genbank (https://www.ncbi.nlm.nih.gov/genbank/; Appendix S1). There are no sequences available for Atlantic Forest species *Sphaenorhynchus bromelicola* and *S*. *palustris*, and therefore were not included in our analyses. We obtained sequences from 41 specimens from the Sphenorhynchini tribe in addition to *Scinax fuscovarius* which was used as an outgroup (Hime et al., 2021; see Appendix S1).

For phylogenetic analysis, we used four molecular markers (see Appendix S1), three mitochondrial genes (12S, 16S, and Cytochrome b—Cytb), and two nuclear genes (Recombination Activating 1—Rag1 and Tyrosinase—Tyr). This led to an alignment of 687 base pairs (bp) for the 12S gene, 1,053 bp for the 16S gene, 299 bp for the Cytb gene, 370 bp for Rag1, and 432 bp for the Tyr gene. The sequences were aligned with the ClustalW algorithm (Sievers et al., 2011) and visually verified in the Geneious v9.1.2.

### 2.2 Phylogenetic analysis

To estimate the best substitution model for each gene segment, we used the Bayesian Information Criterion (BIC, Sullivan & Joyce, 2005) implemented in the jModelTest 2.1.4 program (Darriba et al., 2012). The most suitable models were GTR+I+G for 12S, GTR+I+G for 16S, HKY+I+G for Cytb, K80+G for Rag1, and K80+G for Tyr. To infer the timing of speciation events within the tribe Sphaenorhynchini, we built a species tree in *BEAST using the three mitochondrial and two nuclear genes in BEAST v2.6.3 (Bouckaert et al., 2019). Due to the lack of fossil calibrations for this group, we used the 16S mutation rate of protein proposed by Lemmon et al. (2007) and Lymberakis et al. (2007) (0.28% per lineage per million years) and a mutation rate of Cytochrome b of anurans of 0.957% per lineage per million years (Crawford, 2003). We ran 300 million generations, sampling every 30,000 steps using a tree from the Yule Process prior. We visually evaluated the convergence of the MCMC executions and the effective sample sizes (ESS values ≥200) using the TRACER 1.7 program (Rambaut et al., 2018). The first 10% of sampled genealogies were discarded as burn‐in, and the most credible clade was inferred with TreeAnnotator v2.6.3 (Bouckaert et al., 2019).

### 2.3 Biogeographical analysis

**FIGURE 1:**
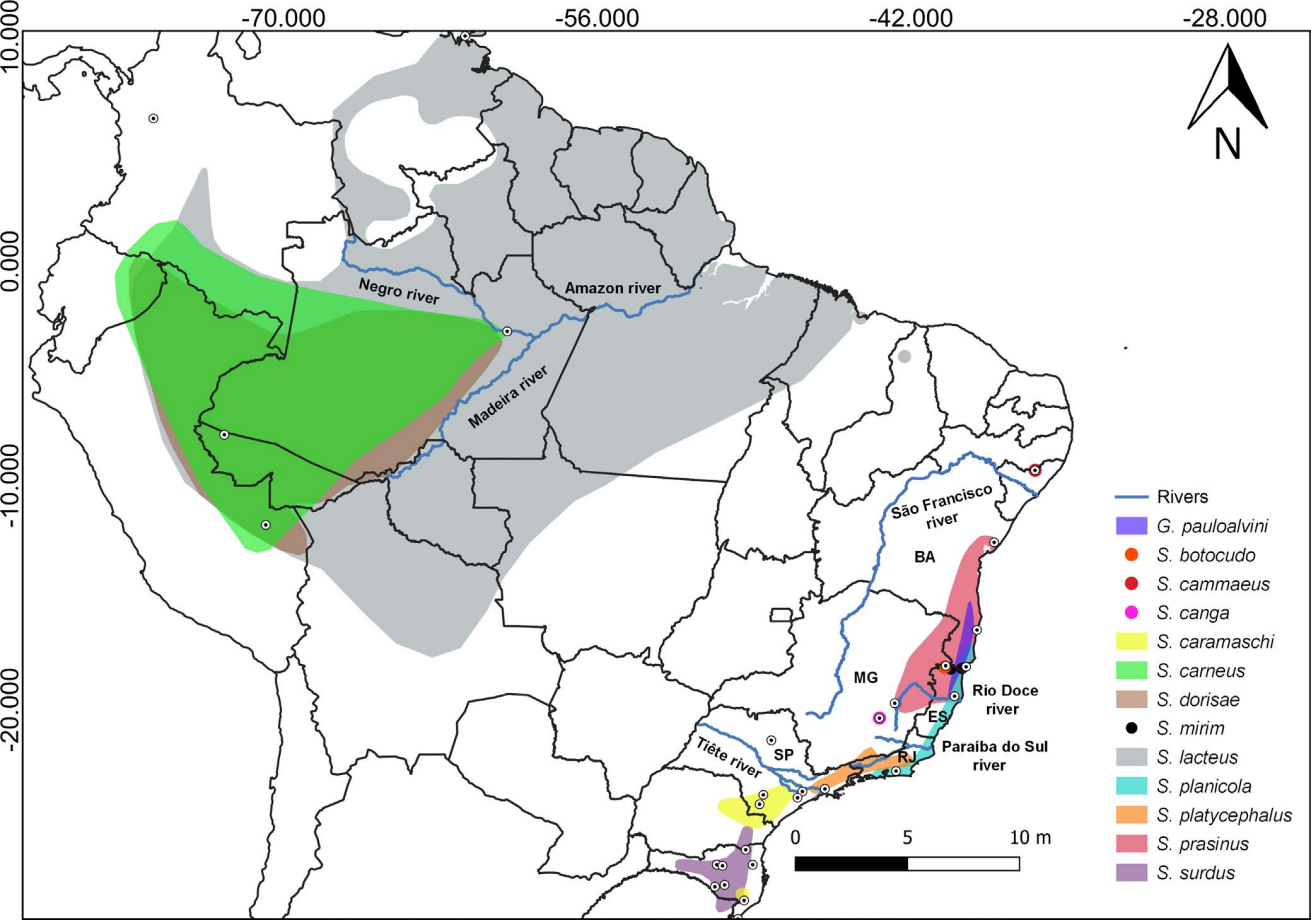
Geographic distribution of the species of the Sphaenorhynchini tribe in South America. White dotted circles: occurrence points + molecular data. Colors represent the range of distribution of each species of the Sphaenorhynchini tribe

The geographical distribution over time of the Sphaenorynchini tribe in South America was estimated with the ‘BioGeoBEARS’ package (Matzke, 2013, 2014) in the R environment (R Core Team, 2020). The variety of Sphaenorynchini species has been subdivided into five geographical areas based on distribution maps available on the IUCN Red List (IUCN, 2020): (a) West Amazonia, (b) East Amazonia, bordered by the Madeira and Negro rivers, (c) South Atlantic Forest, (d) Middle Atlantic Forest, and (e) North Atlantic Forest, bordered by the Tietê and Paraíba do Sul Rivers (Figure 1). The “BioGeoBEARS” package implements Maximum Likelihood (ML) methods that replicate the main premises of the three methods most commonly used in historical biogeography, namely, DEC (Dispersion‐Extinction Cladogenesis; Ree & Smith, 2008), DIVA (Analysis of Dispersion‐Vicariance; Ronquist, 1997), and BayArea (Bayesian Inference from Historical Biogeography for Discrete Areas; Landis et al., 2013). These three methods were originally developed in different structures (Probability for DEC, Parsimony for DIVA, and Bayesian for BayAREA), but they are all represented as probability models in “BioGeoBEARS” to allow direct comparison. The latter two models are therefore not identical to their original formulation and are referred to as DIVALIKE and BAYAREALIKE within “BioGeoBEARS” (Matzke, 2013). Collectively, these models allow for a wide range of processes, including speciation within the area, vicariance, range expansion (dispersion to a new area), and range contraction (extinction in an area). We also tested models with and without founder event speciation, which is incorporated into parameter j. From the model with the lowest Akaike information criterion (AIC), we estimate the probabilities of the ancestral area along the phylogeny (Burnham & Anderson, 2003).

In DEC, the geographic range is allowed to change across a phylogeny through several types of events. Along the branches of a phylogenetic tree (anagenetic evolution), the events allowed are “dispersal” (range expansion by adding an area) and “extinction” (range reduction through extirpation in an area), and these are treated as continuous‐time Markov processes (Matzke, 2014). In general, the three "+ *j*" models significantly improved the fit of the model to the corresponding model without the inclusion of "+ *j*." For Sphaenorynchini, under DEC + *j*, the *j* parameter is always positive, and the *d* and *e* parameters are inferred to be closer to zero. This is an indication that the “D” and “E” processes of the DEC model are unnecessary for explaining the biogeography of Sphaenorynchini. Instead, the data are explained with a much higher probability by a series of founder events. We are aware of the critique of DEC/DEC + j statistical comparisons put forward by Ree and Sanmartín (2018), but we decided to maintain this parameter based on the replies indicating the statistically invalid presented by Klaus and Matzke (2020) and Matzke (2021) about DEC/DEC + j comparisons.

## 3 RESULTS

### 3.1 Phylogeny and divergence times

**FIGURE 2:**
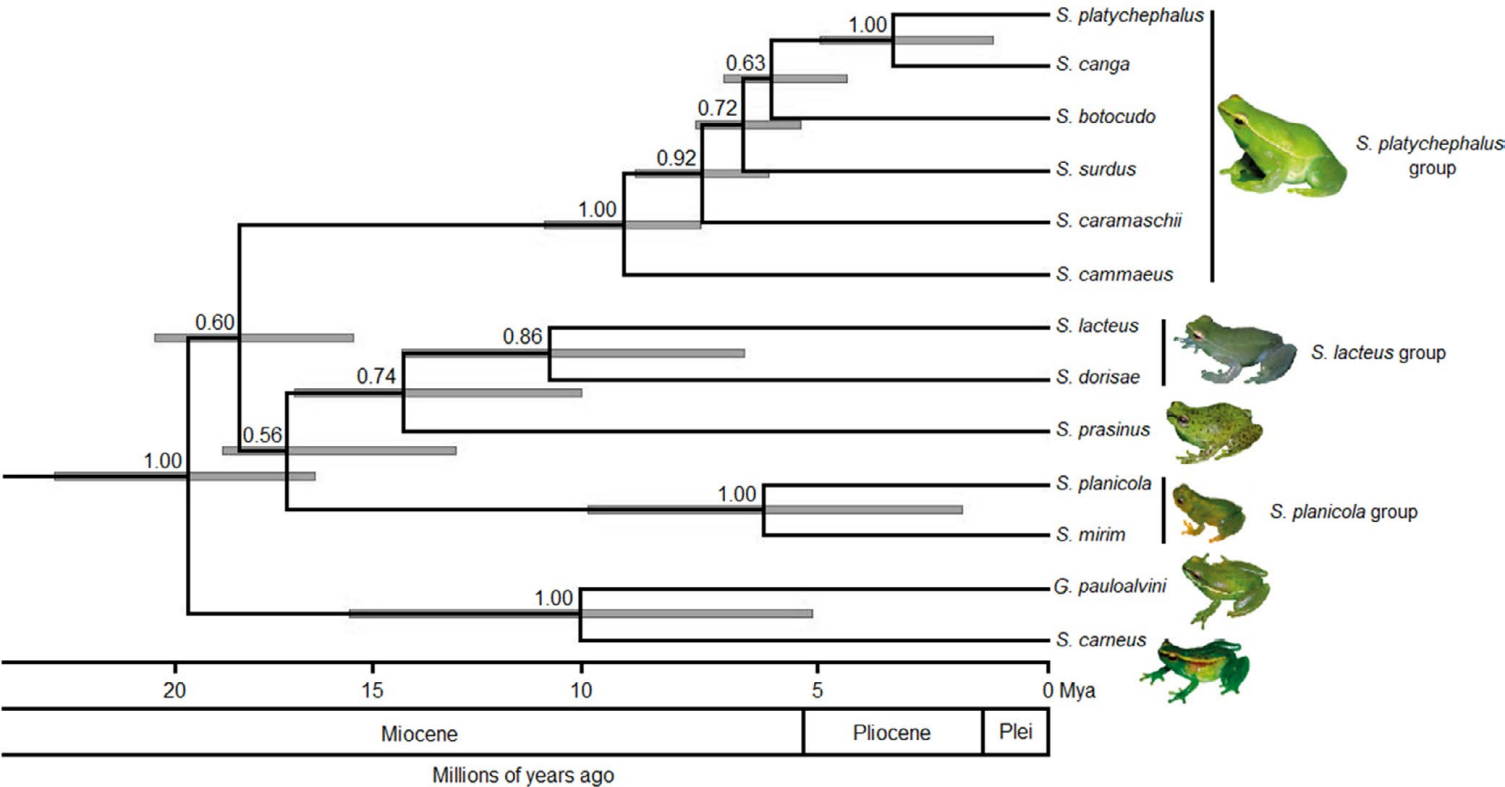
Species tree of the Sphaenorhynchini tribe based on the Bayesian analysis of the 12S, 16S, Cytochrome b, and Tyrosinase genes. Values above the branches indicate posterior probabilities. The scale bar represents the number of substitutions per site. Photos of I.J. Roberto (*S*. *cammaeus* – *S*. *platycephalus* group) and *S*. *prasinus*, F.S.F. Leite (*S*. *canga*), C.E. Costa‐Campos (*S*. *carneus*), T. Grant (*S*. *lacteus* – *S*. *lacteus* group), J.L. Gasparini (*S*. *mirim – S*. *planicola* group), and M.T. Rodrigues (*G*. *pauloalvini*)

In the Bayesian analysis, 58% of the nodes were strongly supported (Figure 2). Most speciation events that reproduce the current diversity occurred between 19 and 6 million years ago, and the greatest diversification was observed from the middle Miocene to the beginning of the Pliocene. Three principal clades (*Sphaenorhynchus platycephalus* group, *S*. *lacteus* group, and *S*. *planicola* group), all monophyletic, were recuperated from the *Sphaenorhynchus* lineages (Figure 2).

The most recent common ancestor (MRCA) of Sphaenorhynchini was estimated at 19.23 Mya (HPD 95%: 16.41–22.23 Mya), between the end of the Oligocene and the beginning of the Miocene, with strong support in Bayesian phylogeny (PP = 1.00). Relatively shortly after the initial division, between the end of the Paleogene and the beginning of the Neogene, the clade containing *G*. *pauloalvini* and *S*. *carneus* diverged from all other Sphaenorhynchini (19.23 Mya; 95% HDP: 16.41–22.23 Mya) in different ecoregions, Amazonia and Atlantic Forest. The MRCA of these two species was estimated to be 10.49 Mya, at the end of the Miocene (95% HDP: 5.03–15.65 Mya). At about the same time, the clade containing the *S*. *platycephalus* group (*S*. *platycephalus*, *S*. *canga*, *S*. *botocudo*, *S*. *surdus*, *S*. *caramaschi*, and *S*. *cammaeus*) diverged from the other *Sphaenorhynchus* 18.09 Mya also at the beginning of the Miocene (95% HDP: 15.56–19.99 Mya). About one million years later, there was a new divergence, giving rise to *S*. *planicola* group (*S*. *planicola* and *S*. *mirim*) about 17.08 Mya (95% HDP: 13.26–218.48 Mya). In the middle Miocene, a divergence was estimated at 14.47 Mya (95% HDP: 10.46–16.86 Mya), giving rise to two new clades, separating *S*. *prasinus* from the Amazonia clade (*S*. *lacteus* group). This was followed by a new division, separating *S*. *lacteus* and *S*. *dorisae* during the end of Miocene 11.19 Mya (95% HDP: 6.83–14.47 Mya). Within the *S*. *platycephalus* group, five diversification events can be observed between the Miocene and the Pliocene (Figure 2). The initial division of existing species in this group was estimated during the Miocene period (9.52 Mya; 95% HDP: 7.79–11.29 Mya). In this same period, there was a divergence between the two species of the *S*. *planicola* group (*S*. *mirim* and *S*. *planicola*), dated 6.4 Mya (95% HDP: 1.96–10.32 Mya).

### 3.2 Ancestral area estimates

**FIGURE 3:**
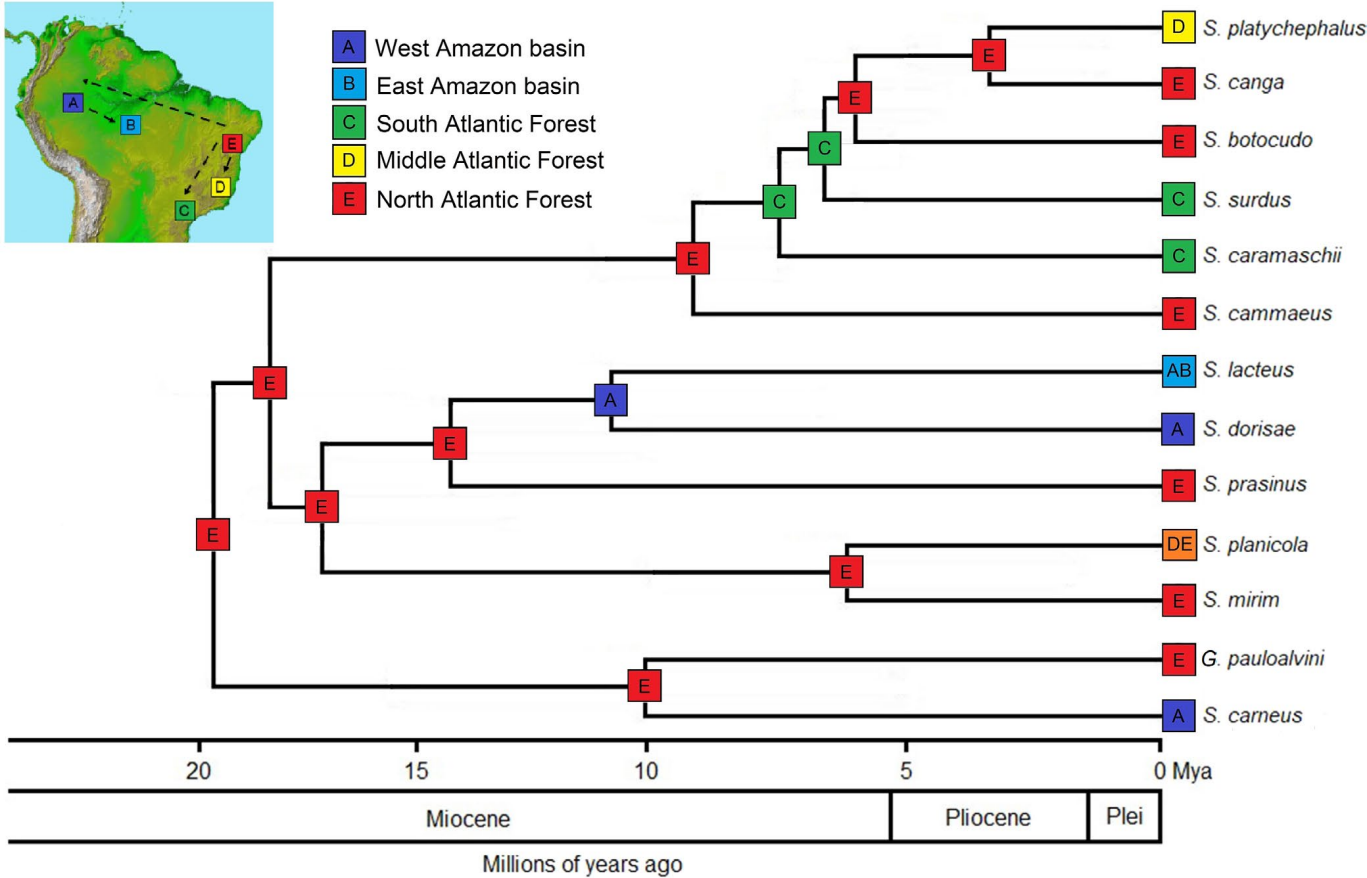
Inferred biogeographic scenario for the species of *Sphaenorhynchus* and *Gabohyla* through the best‐fit model recovered by BioGeoBEARS (DEC+j). The main biogeographic areas defined based on the Sphaenorhynchini distribution are shown on the map as follows: West Amazon basin (dark blue), East Amazon basin (light blue), South Atlantic Forest (green), Middle Atlantic Forest (yellow) and North Atlantic Forest (red). The arrows on the map represent the dispersion events inferred to occur along the branches. See Figure 2 for posterior probabilities of the clades

The DEC model, through a founding event (+ *j*), was the most adequate to the data (AIC = 54.70; Table 1). Our results indicate that the first Sphaenorhynchini diversification event occurred in the North Atlantic Forest, about 19 million years ago, at the beginning of the Miocene (also supported by models without the “+J” parameter, Figure 3). In general, the three “+J” models improved model‐fit significantly for the corresponding model without the inclusion of “+J.” Similar scenarios were also obtained by the DIVALIKE+J model, which was the second‐best model inferred from our data by BioGeoBEARS (AIC = 56.01; Table 1).

**TABLE 1:** Comparison of the BioGeoBEARS model for Sphaenorynchini based on the log‐likelihood (LnL) and the Akaike information criterion (AIC); *N*, parameters number; *d*, dispersion rate; *e*, extinction rate; *J*, the relative probability of speciation between founding events. The best model shown in bold

Model	LnL	*N*	*d*	*e*	*J*	AIC
DEC	−31.318	2	0.00828	0.00160	0.00	66.64
**DEC+j**	**−24.350**	**3**	**0.00309**	**0.00000**	**0.16**	**54.70**
DIVALIKE	−30.844	2	0.01114	0.00000	0.00	65.69
DIVALIKE+j	−25.003	3	0.00400	0.00000	0.12	56.01
BAYAREALIKE	−34.798	2	0.00759	0.07836	0.00	73.60
BAYAREALIKE+j	−26.144	3	0.00324	0.00000	0.12	58.29

According to DEC+J model, two distinct dispersion events occurred with species between forest ecoregions, Amazonia and Atlantic Forest. The initial dispersions occur at the beginning of the Miocene, when there was radiation from the common ancestor and an event restricted *S*. *carneus* to the West Amazonia, and *G*. *pauloalvini* in the Atlantic Forest, specifically between the North of the Paraíba do Sul River and the Jequitinhonha River valley (Figure 3). In the middle Miocene, there was also a new divergence, giving rise to two new clades. Thus, new radiation of lineages from the Atlantic Forest to the Amazonia occurs. After this diversification, an event occurred and restricted *S*. *dorisae* to the West of the Madeira River, and *S*. *lacteus* was widely distributed in the Amazonia (Figure 3).

The clade with Atlantic Forest species diversified around 18 Mya, restricting *S*. *mirim* to the North of the Rio Doce River, on the border of the states of Bahia and Espírito Santo, and *S*. *planicola* to the coastal region of the South of the Bahia state to the South of the Rio de Janeiro state. The second diversification event in the Atlantic Forest was estimated for the middle Miocene (about 14 million years ago), restricting *S*. *prasinus* to the North of the Paraíba do Sul River. However, most diversifications of the Atlantic Forest lineages occurred from the late Miocene until the beginning of the Pliocene, when five different events occurred between 9 and 6 million years ago, restricting *S*. *cammaeus* to Northeast Brazil; subsequently, *S*. *caramaschi* and *S*. *surdus* to the South of the Tietê River and divided *S*. *planicola* to the coastal region of the Atlantic Forest, specifically, from the South of the Bahia state to the South of the Rio de Janeiro state; and *S*. *mirim* restricting it to the North of the Espírito Santo state, on the border with the Bahia state. At the end of the Miocene, other diversification events took place, restricting *S*. *botocudo* also to the North of the Rio Doce River and then the most recent lineages to diversify were *S*. *canga* (restricted to the North of the Paraíba do Sul River) and *S*. *platycephalus* (occurring from the North of the Tietê River to the South of the Paraíba do Sul River), occurring in the Pliocene (about 3,5 million years ago) (Figure 3).

## 4 DISCUSSION

This is the first large‐scale study to include a calibrated phylogeny of Sphaenorhynchini, presenting important information about the evolution of the tribe and the species diversification. Based on the divergence dates inferred by our analyses, the diversification of the 13 species of Sphaenorhynchini from the Amazonia and Atlantic Forest lineages coincided with the main climatic and geological factors that shaped the Neotropical landscape during the Cenozoic. The MRCA of the Sphaenorhynchini species emerged in the North of the Atlantic Forest and migrated to the Amazonia in different dispersion events that occurred during the connections between these ecoregions, as hypothesized. Overall, we suggest that biogeographic historical of Sphaenorhynchini have resulted from a combination of repeated range expansion and contraction cycles concurrent with climate fluctuations and dispersal events between the Atlantic Forest and Amazonia.

In our species tree, we recovered three characteristic clades that we recognize as groups of species (*S*. *lacteus*, *S*. *planicola*, and *S*. *platycephalus* group), with *S*. *carneus* and *G*. *pauloalvini* the sister taxa of all other species of the tribe and a high support value (100%) for the clade, differently from that found by Araújo‐Vieira et al. (2019, 2020), which did not include *S*. *carneus* as a sister species to the rest of the tribe with low support value. According to Araujo‐Vieira et al. (2019), *Sphaenorhynchus pauloalvini* appears as a sister taxon of all other species of the genus, as it has several character states that differs it from the other species of *Sphaenorhynchus*. However, these differences in the inferred phenotypic synapomorphies result from limitations in the taxonomic sampling of previous studies (Araujo‐Vieira et al., 2015; Bokermann, 1973). Therefore, Araujo‐Vieira et al. (2020) reviewed the synapomorphies resulting from the phylogenetic analyzes made by Araujo‐Vieira et al. (2019) and found some inconsistencies in the optimizations in some character states, and based on these phenotypic synapomorphies, they considered *S*. *pauloalvini* a morphologically unique species within the genus *Sphaenorhynchus*, and for this reason, they chose the new monotypic genus *Gabohyla* for this species, even obtaining a low support value (65%) for the clade of *G*. *pauloalvini* in the parsimony analysis.

The differences found among our study and Araújo‐Vieira et al. (2019, 2020) in relation to the species tree may be due to the different approaches used in the studies, where we used Bayesian phylogenetic analysis and the last used the parsimony method. Maximum Parsimony (MP) is a method that tries to minimize the number of mutations because it considers that one mutation is more likely than two. It is a discrete method and does not use probabilistic evolution models (Garcia, 2007). The major problem with this method is that it fails to take into account many sequences evolution factors (e.g., reversals, convergence, and homoplasy). Thus, the deeper the divergence times the more likely these methods will lead to erroneous or poorly supported groupings. Bayesian Inference (BI) is based on a posteriori probability, using an a priori probability and generating a phylogenetic tree according to the data. Supposedly infers trees with high support for clades, provides a distribution of trees that allows the choice of hypotheses (trees) with greater posterior probability (Lewis et al., 2005; Mar et al., 2005). One of the most appealing aspects of Bayesian phylogenetic inference is its presentation and comparison of multiple optimal hypotheses. While a MP attempts to produce the shortest topologies, BI produces a range of solutions, each with a corresponding overall posterior probability as well as comparable node support values for alternative topologies within each tree hypothesis (Li, 1996; Mau et al., 1999). Some studies have also suggested that BI trees have a higher resolution than MP (Spencer & Wilberg, 2013). Thus, for this reason, we decided not to consider the new genus (*Gabohyla*) proposed by Araujo‐Vieira et al. (2020), and from now on we will attribute *G*. *pauloalvini* as belonging to the genus *Sphaenorhynchus* (*S*. *pauloalvini*). The rest of the species tree topology in our analyzes corroborates Araujo‐Vieira et al. (2019) and most conflicts are restricted to relationships within the *S*. *platycephalus* group, where there are two species for which we do not have genetic data (*S*. *bromelicola* e *S*. *palustris*). Nonetheless, this result highlights the need for further investigations about this relationship, given that our analysis (species tree) is based on robust coalescent models, and thus, the inclusion of more individuals and more genetic markers may reveal these relationships more adequately.

Based on the divergence dates inferred by the Bayesian analysis, the diversification of the existing species of *Sphaenorhynchus* from the Amazonia and Atlantic Forest lineages corroborate the main climatic and geological factors that shaped the Neotropical landscape during the Cenozoic (Linder, 2008; Perret et al., 2013; Rull, 2011a). The MRCA of the Sphaenorhynchini species emerged in the north of Atlantic Forest and migrated to the Amazonia in different dispersion events that occurred during the connections between these ecoregions. After the Cretaceous–Paleogene extinction event, paleoclimatic and palynological analyses (e.g., Costa, 2003; Ledru, 1993; Micheels et al., 2007; Ortiz‐Jaureguizar & Cladera, 2006; Sobral‐Souza et al., 2015) indicate that the climate of South America was humid and hot during much of its range in the Paleogene, due to the PETM (Paleocene–Eocene Thermal Maximum). This climate would have promoted forest development across the continent, allowing the Amazonia and Atlantic Forests to be connected (Costa, 2003; de Oliveira et al., 1999; Patton et al., 1997; Wang et al., 2004; Willis, 1992) through different biogeographic routes (see Por, 1992). However, during the Eocene–Oligocene (~34 Mya) the climate began to undergo sudden changes due to the isolation of the Antarctic (Carter et al., 2017; Goldner et al., 2014; Kvasov & Verbitsky, 1981), causing global cooling. The climatic fluctuations continued through the Oligocene and Miocene (Graham et al., 2010; Jaramillo et al., 2010; Zachos et al., 2001) changing the composition of vegetation worldwide (Meseguer et al., 2015) and probably caused the contraction and the rupture of previously continuous tropical forest areas (Jaramillo et al., 2010), influencing the diversification of groups in different ecoregions (Antonelli et al., 2010; Hughes et al., 2013). These patterns suggest multiple connections between the Atlantic Forest and the Amazonia over time, promoting the exchange of fauna in both, abandoning the idea of a major migratory event in a single direction. Our reconstruction supports this hypothesis, suggesting that the beginning of Sphaenorhynchini diversification between an Amazonia and Atlantic clade occurred in the middle Miocene at around 14 Mya and the second diversification around 10 Mya with credibility intervals that range up to the beginning of the Pliocene. Thus, the presence of *Sphaenorhynchus* in the Amazonia is probably the result of two different dispersion events that occurred during these connections, in agreement with the different findings of multiple connections between these two regions.

Based on the multiple connections between the Atlantic Forest and the Amazonia over time and from biogeographical reconstructions, we suggest that the ancestor area to the origin of Sphaenorhynchini probably occupied a wide geographic area in Eastern South America, which today is the Northeast of the Atlantic Forest (Area E; Figure 3). This is because almost all the tribe species belong to only one defined geographical area, except *S*. *lacteus* (belonging to two areas, A and B) and *S*. *planicola* (belonging to two areas, D and E). However, we assume that Sphaenorhynchini spread through forested areas during the Oligocene (29 Mya), a period when forested areas probably extended from the Amazonia to the Atlantic Forest. The contraction of forest areas during the Oligocene until Miocene may have isolated a lineage in the North Atlantic Forest, which diverged, giving rise to *G*. *pauloalvini*, a data deficient species with a distribution limited (de Freitas et al., 2009; Peixoto & Pimenta, 2004). Likewise, it isolated another lineage in the West Amazonia basin, giving rise to *S*. *carneus*, a species of limited distribution by the Amazon and Madeira Rivers (Azevedo‐Ramos et al., 2004; de la Riva et al., 2000) (Figure 1). Also, in the Miocene, diversification of other lineages descending from the MRCA *Sphaenorhynchus* began.

The idea of forest corridors connecting the Eastern Amazonia and the Northeast Atlantic Forest (Ledo & Colli, 2017; Melo Santos et al., 2007; Rizzini, 1963) are corroborated with the biogeographic standards that we found for species of the tribe Sphaenorhynchini. Although the “Dry Diagonal of Open Formations” has limited migration between the Amazonia and Atlantic Forest, which are important in the diversification of amphibians and reptiles (Castroviejo‐Fisher et al., 2014; Fouquet, Loebmann, et al., 2012; Fouquet, Recoder, et al., 2012; Prates et al., 2016; Thomé & Carstens, 2016; Thomé et al., 2016), gallery forests and more humid portions, usually in high altitude areas, maintained some connectivity between forest ecoregions (Costa, 2003; Fine & Lohmann, 2018; Ledo & Colli, 2017; Sobral‐Souza et al., 2015). For example, fossils, paleopalinological data, and speleothems from the Caatinga ecoregion in Northeastern Brazil indicate that in the past xeric vegetation was replaced by species of tropical forest trees, due to higher levels of precipitation (Auler & Smart, 2001; Auler et al., 2004; Cartelle & Hartwig, 1996; Czaplewski & Cartelle, 1998; de Oliveira et al., 1999; Wang et al., 2004). Furthermore, the existence of the Caatinga enclaves’ moist forests, which are forest entrances within the semi‐arid vegetation of the Caatinga ecoregion, forming islands of humid forest (Andrade‐Lima, 1982) containing a mixture of species with Amazonia and Atlantic affinities supports the hypothesis of forest corridors (Mângia et al., 2018). Other potential factors also influenced the diversification patterns, such as geological history (tectonic movements and mountain orogenesis), which had a profound consequence for the origin and evolution of Neotropical biodiversity by increasing and breaking of biogeographic barriers (Antonelli et al., 2009; Moritz et al., 2000). For example, along the Neogene (24–2 Mya), tectonic events, such as the Andean uplift, affected the climate of South America, which in turn drastically changed the Amazonia landscape with the formation of the Pebas system, a large marsh that separated the Western and Eastern South America and the formation of the Acre System (beginning of the formation of the Amazon River), delimiting the Amazon basin in the Northern and Southern parts, promoting the evolution of new lineages (Antonelli & Sanmartín, 2011; Hoorn, Wesselingh, ter Steege, et al., 2010; Insel et al., 2010; Latrubesse et al., 2010; Morley, 2000), likewise for Sphaenorhynchini.

Given the distribution of Amazonia species (*S*. *carneus*, *S*. *lacteus*, and *S*. *dorisae*), the change in the aquatic landscape probably influenced the diversification of this lineage of *Sphaenorhynchus*, since the diversification between *S*. *dorisae* and *S*. *lacteus* (~11 Mya) corresponds to the period in which the drainage system of the Amazon basin was being formed (Hoorn, Wesselingh, ter Steege, et al., 2010). Thus, it may have restricted *S*. *dorisae* in the Western Amazonia, between the Madeira and Negro Rivers while *S*. *lacteus* was widely distributed, associated with the entire Amazonia basin, also occurring in transition zones between the Amazonia and the Cerrado (see Silva et al., 2020) and in gallery forests, between Cerrado and Caatinga, in Northeastern Brazil (see Benício et al., 2011). The wide distribution of this species is a curious fact and may be due to its ancient diversification, but only with a phylogeographic study covering the entire distribution of this species, it will be possible to understand which historical events influenced the current geographical distribution.

Following the same pattern, in the Atlantic Forest is evident that diversity is highly structured along a North–South gradient, and that rivers probably played an important role in this divergence (Behling, 1997; Jackson, 1978; Ledru et al., 2005; Oliveira‐Filho & Fontes, 2000; Pellegrino et al., 2005). A large number of phylogeographic studies with taxa from this ecoregion have identified some main barriers to gene flow, through the separation of closely related sister taxa, such as a break in São Paulo state, close to the Tietê River valley; breaks in Minas Gerais state, close to the Paraíba do Sul River, Rio Doce River and Jequitinhonha River valley and a break Northeastern Brazil, close to the São Francisco River valley (see Amaro et al., 2012; Batalha‐Filho et al., 2012; Carnaval et al., 2009, 2014; Costa, 2003; D’Horta et al., 2011; Grazziotin et al., 2006; Pellegrino et al., 2005; Pirani et al., 2020; Resende et al., 2010; Thomé et al., 2010; Valdez & D'Elía, 2013). It is possible to observe some of these barriers in the distribution of Sphaenorhynchini species, such as, for example, the Paraíba do Sul River valley in Rio de Janeiro and Minas Gerais states and the Rio Doce River in Espiríto Santo and Minas Gerais states, which together, is the current limit from the distributions of *S*. *canga* (see Araujo‐Vieira et al., 2015), *S*. *prasinus* (see da Silva et al., 2013), *S*. *pauloalvini* (see Freitas et al., 2009), and *S*. *mirim* (see Caramaschi et al., 2009). The Tietê River region in São Paulo state, is the current limit of the *S*. *caramaschi* distribution (see Melo et al., 2018), *S*. *surdus* (see Toledo et al., 2007) and may have played an important role in the divergence of these species from the ancestor of the other species of Sphaenorhynchini.

Despite the role of rivers as barriers in Amazon and in Atlantic Forest, recent studies have associated that the climatic fluctuations of the Pleistocene induced the fragmentation of the forest formations (Cabanne et al., 2008; Carnaval et al., 2014; Thomé et al., 2010), isolating limited‐dispersal organisms. The remaining forest fragments would be isolated and, in these forest refuges, new species would emerge from the widely distributed ancestral species (Carnaval & Moritz, 2008; Martins, 2011). The Pleistocene refuges (Carnaval et al., 2009; Haffer, 1969, 1997; Vanzolini & Williams, 1981) have been used and reviewed as a scenario that explains what caused the increase in the diversity and richness rate of these environments (Bush & Oliveira, 2006; Connor, 1986; Garzón‐Orduña et al., 2015). According to this hypothesis, climatic oscillations during the Last Glacial Maximum (LGM) would have maintained a stable climate and forest environments in some areas, thus serving as a source of refuge for recolonization (Arruda et al., 2018; Sobral‐Souza & Lima‐Ribeiro, 2017). Given the probable geographic distribution of endemic species of *Sphaenorhynchus*, which diverged between 29 and 3.5 Mya, during the Oligocene, mainly during the Miocene (which was the main period of origin of most lineages) and Pliocene in the Atlantic Forest, we can observe patterns of similar occurrences with the refuge areas proposed by Carnaval and Moritz (2008) and Carnaval et al. (2009). For example, *S*. *cammaeus* is known only for one location in the Pernambuco refugium (see Roberto et al., 2017); other species are limited to the Bahia refugium, such as *S*. *mirim*, *S*. *botocudo*, and *G*. *pauloalvini* (see Caramaschi et al., 2009; de Freitas et al., 2009) and *S*. *caramaschii* occurring in the São Paulo refugium (see Melo et al., 2018). However, the divergence times between species are older than the LGM. Thus, the Miocene climate changes may have played a central role in the simultaneous origin of these taxa, showing that some of these forest fragments remained relatively stable for a much longer period than that proposed by Carnaval and Moritz (2008), thus serving as a successive refuge in the climatic cycle. Phylogeographic studies, covering the area of occurrence of all species of the Sphaenorhynchini tribe, with extensive sampling, will clarify and allow a better understanding of these patterns.

## AUTHOR CONTRIBUTIONS

**Elvis Almeida Pereira:** Conceptualization (equal); Data curation (equal); Formal analysis (equal); Methodology (equal); Visualization (equal); Writing – original draft (equal); Writing – review & editing (equal). **Karoline Ceron:** Formal analysis (equal); Investigation (equal); Methodology (equal); Software (equal); Writing – original draft (equal); Writing – review & editing (equal). **Hélio Ricardo da Silva:** Investigation (equal); Project administration (equal); Resources (equal); Supervision (equal); Writing – review & editing (equal). **Diego José Santana:** Conceptualization (equal); Formal analysis (equal); Funding acquisition (equal); Investigation (equal); Project administration (equal); Resources (equal); Software (equal); Supervision (equal); Writing – original draft (equal); Writing – review & editing (equal).

## Supporting information

Supplementary Material

## Data Availability

The alignments used in this study is available at Github Digital Repository at https://github.com/Rhinella85/Biogeography‐of‐Sphaenorhynchus
