# Supplementary material for: The dispersal between Amazonia and Atlantic Forest during the Early Neogene revealed by the biogeography of the treefrog tribe Sphaenorhynchini (Anura, Hylidae)
Source: Ecol Evol. 2022 Apr 1;12(4):e8754. doi: 10.1002/ece3.8754 (PMC8975791; doi:10.1002/ece3.8754)
Supplement: Supplementary file 1 — Supplementary Material [file ECE3-12-e8754-s001.docx]

**Supplementary Material 1.** Samples, collection numbers, localities and GenBank access numbers of mitochondrial and nuclear sequences used in this study. (-) genes not sequenced. Institutional abbreviations follow Sabaj (2016). CRR = Camila R. Rievers field number. JMP = José M. Padial field number. MVS = Magno V. Segalla field number. MTR and ITH = Miguel T. Rodrigues field number. SR = Steffen Reichle field number.

| **Samples** | **Species** | ***12S* and *16S*** | ***Cytochrome b*** | ***Recombination Activating*** ***1*** | ***Tyrosinase*** | **Locality** |
| --- | --- | --- | --- | --- | --- | --- |
| **Outgroup** |  |  |  |  |  |  |
| SR 87 | *S. fuscovarius* | MK266760 | MK266612 |  | MK266695 | Bolivia: Tarija |
| **Ingroup *Sphaenorhynchus*** |  |  |  |  |  |  |
| MNRJt 483 | *S. botocudo* | MK266724 | MK266587 | MK266614 | MK266670 | Brazil: State of Espírito Santo, Municipality of Mucurici, Lagoa Nova |
| UFMG 20767 | *S. botocudo* | MK266723 | MK266586 | - | - | Brazil: State of Bahia, Municipality of Mucuri |
| UFMG 20768 | *S. botocudo* | MK266722 | MK266585 | - | - | Brazil: State of Bahia, Municipality of Mucuri |
| ZUFG 5360 | *S. botocudo* | MK266725 | MK266588 | - | - | Brazil: State of Bahia, Municipality of Porto Seguro |
| MACN-He 48851 | *S. cammaeus* | MK266727 | MK266590 | MK266616 | MK266672 | Brazil: State of Alagoas, Municipality of Quebrangulo, Rebio Pedra Talhada |
| MACN-He 48852 | *S. cammaeus* | MK266726 | MK266589 | MK266615 | MK266671 | Brazil: State of Alagoas, Municipality of Quebrangulo, Rebio Pedra Talhada |
| MNRJ 56336 | *S. canga* | MK266728 | MK266591 | MK266617 | MK266673 | Brazil: State of Minas Gerais, Municipality of Mariana |
| CFBH 6876 | *S. caramaschii* | MK266734 | MK266594 | MK266621 | - | Brazil: State of São Paulo, Municipality of Ribeirão Branco |
| CFBH 10325 | *S. caramaschii* | MK266732 | MK266592 | MK266619 | MK266675 | Brazil: State of Santa Catarina, Municipality of Treviso |
| CFBH 11285 | *S. caramaschii* | MK266733 | MK266593 | MK266620 | MK266676 | Brazil: State of São Paulo, Municipality of Ribeirão Branco |
| ITH 0626 | *S. caramaschii* | MK266737 | MK266596 | MK266623 | MK266678 | Brazil: State of São Paulo, Municipality of Buri |
| MCP 11531 | *S. caramaschii* | MK266739 | - | MK266625 | MK266680 | Brazil: State of Rio Grande do Sul, Municipality of Torres |
| MCP 11542 | *S. caramaschii* | MK266738 | - | MK266624 | MK266679 | Brazil: State of Rio Grande do Sul, Municipality of Torres |
| MCP 11980 | *S. caramaschii* | MK266729 | - | - | - | Brazil: State of Santa Catarina, Municipality of São Bento do Sul |
| MCP 11981 | *S. caramaschii* | MK266730 | - | - | - | Brazil: State of Santa Catarina, Municipality of São Bento do Sul |
| MCP 12558 | *S. caramaschii* | MK266731 | - | MK266618 | MK266674 | Brazil: State of Santa Catarina, Municipality of Blumenau, Nova Rússia |
| MZUSP 134240 | *S. caramaschii* | MK266735 | - | - | - | Brazil: State of São Paulo, Municipality of Embu das Artes |
| MZUSP 134698 | *S. caramaschii* | MK266736 | MK266595 | MK266622 | MK266677 | Brazil: State of São Paulo, Municipality of Juquitiba |
| JMP 2091 | *S. carneus* | MK266740 | MK266597 | MK266626 | MK266681 | Colômbia: Amazonas Department, Los Lagos, Várzea Grove |
| JMP 2094 | *S. carneus* | MK266741 | MK266598 | MK266627 | MK266682 | Colômbia: Amazonas Department, Los Lagos, Várzea Grove |
| CFBH 15721 | *S. dorisae* | MK266742 | MK266599 | MK266628 | MK266683 | Brazil: State of Acre, Municipality of Rodrigues Alves |
| MJH 46 | *S. dorisae* | AY843766 | AY844011 | AY844526 | AY844187 | Brazil: State of Amazonas, Municipality of Manaus, Lago Janauari |
| CFBH 15725 | *S. lacteus* | MK266743 | MK266600 | MK266629 | MK266684 | Brazil: State of Acre, Municipality of Rodrigues Alves |
| USNM 152136 | *S. lacteus* | - | AY844012 | AY844527 | AY844188 | Peru: Madre de Dios, 30 km SSW Puerto |
| USNM 268930 | *S. lacteus* | AY549367 | - | - | - | Peru: Madre de Dios, Tambopata Reserve |
| UWIZM 4635 | *S. lacteus* | MK266744 | - | - | - | Trinidad and Tobago: Trinidad, Icacos |
| MNRJt 494 | *S. mirim* | MK266745 | MK266601 | MK266630 | MK266685 | Brazil: State of Espírito Santo, Municipality of Mucurici, Fazenda Matutina |
| CFBH 22920 | *G. pauloalvini* | MK266747 | MK266603 | MK266632 | MK266687 | Brazil: State of Espírito Santo, Municipality of Linhares, Floresta Nacional de Goytacazes |
| CFBH 22925 | *G. pauloalvini* | MK266748 | - | - | - | Brazil: State of Espírito Santo, Municipality of Linhares, Floresta Nacional de Goytacazes |
| MTR 12098 | *G. pauloalvini* | MK266749 | MK266604 | MK266633 | MK266688 | Brazil: State of Espírito Santo, Municipality of Linhares, Floresta Nacional de Goytacazes |
| MTR 12116 | *G. pauloalvini* | MK266750 | MK266605 | MK266634 | MK266689 | Brazil: State of Espírito Santo, Municipality of Linhares, Floresta Nacional de Goytacazes |
| MNRJ 39704 | *S. planicola* | MK266751 | MK266606 | - | MK266690 | Brazil: State of Rio de Janeiro, Municipality of Maricá |
| CFBH 10573 | *S. platycephalus* | MK266746 | MK266602 | MK266631 | MK266686 | Brazil: State of São Paulo, Municipality of Bairro Alto |
| USNM 243667 | *S. platycephalus* | DQ380388 | - | - | - | Brazil: State of São Paulo, Municipality of Salesópolis, próxima a Estação Biológica de Boracéia, Serra do Mar |
| CRR 70 | *S. prasinus* | MK266754 | MK266608 | MK266636 | MK266693 | Brazil: State of Minas Gerais, Municipality of Marliéria, Parque Estadual do Rio Doce |
| MZUFBA 6756 | *S. prasinus* | MK266752 | - | - | MK266691 | Brazil: State of Bahia, Municipality of Mata de São João, Fazenda Camurujipe |
| MZUFBA 6758 | *S. prasinus* | MK266753 | MK266607 | MK266635 | MK266692 | Brazil: State of Bahia, Municipality of Mata de São João, Fazenda Camurujipe |
| CFBHt 5536 | *S. surdus* | MK266756 | MK266610 | MK266638 | - | Brazil: State of Santa Catarina, Municipality of Lebon Regis |
| CFBH 8546 | *S. surdus* | MK266755 | MK266609 | MK266637 | MK266694 | Brazil: State of Santa Catarina, Municipality of Lages |
| MVS 399 | *S. surdus* | MK266757 | - | - | - | Brazil: State of Santa Catarina, Municipality of Santa Cecília |
| UFRGSt 404 | *S. surdus* | MK266758 | - | - | - | Brazil: State of Santa Catarina, Municipality of Campo Belo do Sul |
